# Editorial: Chromosomal Evolution in Plants

**DOI:** 10.3389/fpls.2021.726330

**Published:** 2021-07-29

**Authors:** Martin A. Lysak, Hanna Weiss-Schneeweiss

**Affiliations:** ^1^Central European Institute of Technology (CEITEC), Masaryk University, Brno, Czechia; ^2^Department of Botany and Biodiversity Research, University of Vienna, Vienna, Austria

**Keywords:** chromosomal rearrangements, cytogenomics, karyotype evolution, next generation sequencing, paleogenomics, polyploidy, repeatome, repetitive DNA

Chromosomal evolution is a driving force underlying diversification and speciation in plants. Chromosome numbers and morphology, as well as nuclear DNA amount and composition, are very diverse in plants. During the last few decades, a range of new tools has been developed that allow for better insights into chromosomal and genome evolution in plants, far beyond chromosome counting and establishing karyotype structure. One of the most important tools is next generation sequencing (NGS) technology which has recently revolutionized nearly all biological disciplines. Genome skimming and whole-genome sequencing elevated classical cytogenetics to a new level of modern evolutionary cytogenomics (see also Hu et al., 2020). Comparative plant (cyto)genomics allows for physical localization of DNA sequences on chromosomes, improving whole-genome and chromosome-level assemblies, but also provides a wealth of data on nuclear repeatomes, chromosome structures, mechanisms of chromosomal alterations, and interphase chromosome organization. This Research Topic brings together a collection of research, methodological and review articles advancing our understanding of the role of chromosomal and karyotype evolution in land plant diversification and speciation. It also allows for the exchange of data, ideas, and new hypotheses taking full advantage of novel methodological approaches. The Research Topic consists of 13 original papers, three reviews and one perspective. Altogether these studies fall into one of the four broader intersecting topics: (1) Pathways of karyotype and genome evolution and their role in plant speciation (2) Polyploidy and post-polyploid genome diploidization (3) Paleogenomics and paleogenomes (4) Repeatome and genome size evolution.

## Pathways of Karyotype and Genome Evolution and Their Role in Plant Speciation

Karyotype changes often accompany plant diversification and speciation. Among flowering plant families, the Brassicaceae (mustards, crucifers) has become a prominent model system for studying plant genome and chromosomal evolution due to the availability of abundant genomic resources, such as the high-quality reference genome of *Arabidopsis thaliana*.

The Brassicaceae tribe Aethionemeae, containing a single genus *Aethionema*, is the sister clade to all remaining crucifer lineages. The evolution of the genomes and phylogenetic relationships of this tribe within the Brassicaceae is addressed by Walden et al. Crucifer genomic blocks (GBs) were used to infer syntenies between the genome sequences of *Ae. arabicum* and other crucifer genomes. In contrast to the largely conserved genomic structure of most non-polyploid Brassicaceae lineages, GBs in *Aethionema* were highly rearranged, similar to genomes in the tribe Arabideae. Thus, Arabideae (e.g., the Alpine rock-cress, *A. alpina*) might also have diverged before the other major crucifer lineages.

The Brassicaceae tribe Boechereae comprises about 130 species in nine genera including a number of apomictic species of hybrid origin. Mandáková et al. report here on analyses of the genome structure of seven genera in this tribe using comparative chromosome painting. The ancestral Boechereae genome of *n* = 7 is inferred to be derived from an *n* = 8 genome by descending dysploidy. Although most of the Boechereae genera have a conserved genomic structure, chromosomal rearrangements (most often inversions) have accompanied the divergence of some genera and species within the tribe.

Elucidating chromosomal rearrangements leading to karyotype restructuring requires the use of appropriate genomic tools, as has been elegantly shown for numerous crucifers. Another family for which such tools have now been developed is the Musaceae (bananas). Here, Šimoníková et al. report on the development of chromosome/chromosome-arm specific oligo painting probes that allow for the identification of all chromosomes in *Musa* spp. and for physically anchoring pseudomolecules of the reference genome sequence to individual chromosomes. This has, for the first time, allowed us to gain deeper insight into the evolution of A, B, and S banana genomes, as well as identify chromosomal translocations that have accompanied genome evolution and speciation in *Musa*.

Phylogenetic hypotheses and molecular clock analyses have become essential components of studies on karyotype evolution, as demonstrated by Costa et al. in their analysis of the Amaryllidaceae subfamily Allioideae (e.g., chives, garlics, onions). The three tribes of this subfamily (Allieae, Gilliesieae, and Tulbaghieae) have evolved toward their current intercontinental disjunctions to the Northern Hemisphere, South America, and Southern Africa, respectively. Using a dated phylogeny, together with models of chromosome number evolution and diversification rate analysis, the authors reconstruct the biogeography of the Allioideae to be the result of vicariance due to the break-up of Gondwana; centric fissions are identified as the most likely mechanism of karyotypic diversification in the tribe Gilliesieae.

Chromosome number variation in eukaryotes results from frequent, sometimes very complex, chromosomal rearrangements, which may eventually lead to the reduction of chromosomes, i.e., genetic linkage groups (descending dysploidy). Although such reductions are usually easily detectable in comparative analyses, reconstructing the mechanism underlying these changes is more challenging. Here, Udall et al. investigate the chromosomal rearrangements mediating *n*−1 descending dysploidy in two genera (*Gossypioides* and *Kokia*), sister to the cotton genus (*Gossypium*), using high-quality genome assemblies and Hi-C data. These genome comparisons show that the two descending dysploidies were not mediated by simple recombination between two ancestral chromosomes, but that multiple steps were required to generate the extant genome structures; the evolution of plant chromosomes can be complex and does not necessarily follow the most parsimonious pathways.

As plant genome assemblies are steadily being improved by the increasing read length, we are obtaining a better view of the incidence, scale and structure of chromosomal rearrangements in plant genomes. Inversions are probably the most common chromosomal rearrangements and are ubiquitous across the plant kingdom. Here timely, Huang and Rieseberg review the latest research on chromosome inversions in plant genomes, focusing on the role of inversions in speciation in the presence of gene flow and models of inversion fixation. The authors also discuss sequential inversions and the purported functional link between suppressed recombination and the differentiation of sex chromosomes in plants, such as in the dioecious *Silene* species (Bačovský et al.).

## Polyploidy and Post-Polyploidy Genome Diploidization

Whole genome duplications (WGD) have accompanied the evolution of angiosperms since the beginning. Past and ongoing rounds of polyploidization continue to shape the majority of extant plant genomes.

In a perspective article, Levin offers a personal view on the continued impact of polyploidization on plant diversification. Specifically, the author focuses on mechanisms of polyploid diversification, reasons why WGDs are not followed by immediate diversification thrusts, and on polyploidy going forward. The polyploid wave which began roughly 60 million years ago will, as Levin suggests, continue to rise in the coming millennia, in part due to increasing climate and environmental changes and the impact of other anthropogenic drivers. The proportion of polyploid species might also increase as polyploids are likely to gain an advantage over diploids in changing environments. The increase in polyploidy in the coming millennia might also involve the frequent formation of higher level polyploids and post-polyploid dysploids.

Another process that has a significant impact on plant genome evolution is hybridization. The genomic consequences of such genome mergers alone and in combination with WGD are addressed here in a review paper by Glombik et al. The genomes of newly established interspecific hybrids often undergo dramatic changes, including chromosomal rearrangements, changes in the amount and localization of repetitive DNAs, and gene expression modifications. Successful hybridizations and WGD events are followed by genomic and cytological diploidization events. One of the most significant aspects of this process is establishment of diploid-like chromosome pairing. The authors provide an overview of the current knowledge of genomic changes in interspecific homoploid and alloploid hybrids, focusing on chromosome pairing, and discuss parental genome dominance at various levels of organization in relation to the stability of hybrid genomes.

Domesticated plant species often have polyploid ancestry. Here, Hardigan et al. analyse the genome structure and diversity of the allo-octoploid cultivated strawberry (*Fragaria* × *ananassa*). Comparative genomic analyses show that geographically diverse wild octoploids were diploidized, nearly completely collinear, and retained strong macro-synteny with their diploid progenitor taxa. The conserved genome structure of octoploid *Fragaria* species allows for unimpeded gene flow during repeated cycles of homoploid hybridization without the formation of reproductive barriers or loss of fertility.

## Specialized Chromosomes and Chromosomal Systems

Dioecy is found in 5–6% of angiosperms genera (Vyskot and Hobza, 2004). Some of these taxa have developed differentiated sex chromosomes that have evolved from regular autosomal chromosome pairs. The genus *Silene* (campions, Caryophyllaceae) includes both dioecious and gynodioecious species. Two dioecious species, *Silene latifolia* and *S. dioica*, possess heteromorphic sex chromosomes and an XX/XY sex determination system. Here, Bačovský et al. addressed the origin of X/Y sex chromosomes in *Silene* by mapping an oligo painting probe enriched for X-linked scaffolds to chromosomes of two dioecious and two gynodioecious species. The results suggest shared ancestry of the sex chromosomes in the two dioecious species, accompanied by subsequent extensive chromosomal rearrangements.

Centromeres are chromosomal regions that facilitate faithful chromosome segregation during cell division. The vast majority of plants possess monocentric chromosomes with localized centromeres. Several plant families, however, harbor species or genera with holocentric chromosomes. Cyperaceae (sedges) is an example of one of the families in which holocentricity has been demonstrated and is shown to be associated with frequent dysploid and polyploidization events. Here, Burchardt et al. studied karyotype morphology and genome size evolution in 35 *Rhynchospora* taxa. The reported 22-fold genome size variation and chromosome numbers ranging from 2*n* = 4 to 61 indicate much higher levels of karyotypic diversity than previously believed. Base chromosome number changes were inferred to have occurred in all *Rhynchospora* lineages via dysploidy and polyploidy, driving chromosomal evolution in the genus.

Another genus that harbors species with both monocentric and holocentric chromosomes is parasitic *Cuscuta* (Convolvulaceae, bindweeds). In most organisms, centromeres are determined epigenetically by the presence of the centromere-specific histone variant CENH3. In holocentrics, CENH3 is typically distributed along the entire chromosome length. Here, Oliveira et al. identified CENH3 genes in the holocentric plant *Cuscuta europaea* and found a unique pattern of CENH3 distribution along mitotic chromosomes in this species. Two major CENH3 variants were expressed and co-localized in one to three discrete heterochromatic regions per chromosome that contained the satellite repeat CUS-TR24, whereas the rest of the chromatin appeared to be devoid of CENH3. In contrast, the mitotic spindle microtubules attached at uniform density along the entire chromosome length. The data suggest that CENH3 either lost its function or acts in parallel to an additional CENH3-free mechanism of kinetochore assembly.

## Repeatome and Genome Size Evolution

The tremendous amount of NGS data available allows for detailed insights into plant genome composition and the dynamics of different types of repetitive DNA during diversification and speciation. Apart from a general characterization of the repeat types, NGS data analyses provide the means to conduct comprehensive comparative analyses on the genomic repeat composition of any plant species and their contribution to genome size change, as well as to analyse the evolution of individual repeat families.

Studies involving repeatome dynamics oftentimes focus on model organisms, whereas few provide comprehensive investigations across the genomes of related taxa. Here, McCann et al. analyse the evolution of repeats in a group of 13 closely related diploid species of the genus *Melampodium* (Asteraceae, sunflower family) differing ca. 4.5-fold in genome size despite possessing the same base chromosome number of *x* = 10. Analyses of genome skimming data using the RepeatExplorer pipeline (Novák et al., 2020) determined that patterns of repeat evolution were found to be highly correlated with the phylogenetic position of the species. Evidence was found for strong phylogenetic signal and differential evolutionary rates of major lineages of repeats in the diploid genomes of *Melampodium*.

Several angiosperm families have evolved biomodal karyotypes that consist of chromosomes in two contrasting size classes. One such system is found in the neotropical genus *Eleutherine* (Iridaceae, irises). Both species possess karyotypes with one large and five small chromosome pairs. The large chromosome pair of *E. bulbosa* additionally carries pericentric inversion in permanent heterozygosity in one of its chromosomes. Here, Báez et al. investigate the repeatomes of both species to test whether the permanent chromosomal inversion in the large chromosome influenced the dynamics of repetitive DNA sequences. While the accumulation of repeats differed between large and small chromosomes, the most abundant repeats showed a similar chromosomal distribution in both homologs of the large pair, regardless of the presence of the permanent inversion.

Long terminal repeat (LTR) retrotransposons constitute the most significant part of most plant genomes and the periodical bursts of their activity play an important role in genome size changes, among others. LTR regions are used to estimate the age of retrotransposons, and thus, to date periods of their activity. Here, Jedlicka et al. measure the LTR divergence of thousands of LTR retrotransposons to determine their age and evolutionary dynamics in 15 plant species representing major lineages across Viridiplantae and ranging nearly 10-fold in genome size. The authors hypothesize that gene conversion might have contributed to the higher observed DNA sequence divergence of LTR regions of nested retrotransposon copies compared to the divergence of LTRs of pre-existing retrotransposons into which they had been inserted. The negative correlation between the frequency of gene conversion and the abundance of solo LTRs suggests interference between gene conversion and ectopic recombination.

Another type of tandem repeat that is ubiquitous in nearly all known eukaryotic genomes is found in the telomeres, structures that protect and maintain the ends of linear chromosomes. Telomeric DNA sequences are usually tandemly arranged minisatellites, typically following the formula (TxAyGz)*n*. The review article by Peska and Garcia provides an up-to-date overview of the diversity of plant telomeres following recent discoveries of novel telomeric variants in various plant groups. The authors also provide an overview of methods used for identification of telomeric motifs.

Tandemly repeated ribosomal genes (rRNA genes) are widely used in molecular and cytogenetic studies, including analyses of parental (sub)genomes in homoploid and allopolyploid hybrids. Here, Garcia et al. analyzed 5S rDNA arrays in over 80 homoploid and alloploid hybrid species of different evolutionary ages using graph clustering implemented in the RepeatExplorer pipeline. Comparative analysis of 5S rDNAs in hybrids and their progenitor species allowed for identification of homoeologous 5S rRNA gene families in both evolutionarily young and older taxa, thereby facilitating inferences of their origin. The shapes of cluster graphs robustly reflect the organization and homogeneity of 5S rDNA repeats within each of the parental subgenomes. The authors proposed that this approach, together with cytogenetic analyses, might assist with inferring parental origin of the hybrid taxa.

## Conclusions and Perspectives

This Research Topic presents most recent advances in the field of chromosomal plant evolution, broadly covering most important topics centered around plant genome evolution on all levels of its organization as well as its temporal and spatial aspects. This collection of papers clearly demonstrates that the rapid development of genomic tools and approaches, such as NGS, high throughput data analyses and novel cytogenomic techniques will continue to facilitate the analyses of more wild plant groups and will allow for in-depth analyses of various aspects of chromosomal and genome evolution. Thus, over the coming years we will continue to test old hypotheses in light of novel methodologies and data, and unravel new patterns and phenomena that contribute to genome evolution in plants.

## Author Contributions

MAL and HW-S drafted the manuscript. Both authors revised and approved the final version.

## Conflict of Interest

The authors declare that the research was conducted in the absence of any commercial or financial relationships that could be construed as a potential conflict of interest.

## Publisher's Note

All claims expressed in this article are solely those of the authors and do not necessarily represent those of their affiliated organizations, or those of the publisher, the editors and the reviewers. Any product that may be evaluated in this article, or claim that may be made by its manufacturer, is not guaranteed or endorsed by the publisher.
